# Soft Artificial Synapse Electronics

**DOI:** 10.34133/research.0582

**Published:** 2025-01-28

**Authors:** Md. Rayid Hasan Mojumder, Seongchan Kim, Cunjiang Yu

**Affiliations:** ^1^ Department of Electrical Engineering, The Pennsylvania State University, University Park, PA 16802, USA.; ^2^ Department of Electrical and Systems Engineering, University of Pennsylvania, Philadelphia, PA 19104, USA.; ^3^ Department of Electrical and Computer Engineering, University of Illinois, Urbana-Champaign, Urbana, IL 61801, USA.; ^4^ Department of Materials Science and Engineering, University of Illinois, Urbana-Champaign, Urbana, IL 61801, USA.; ^5^ Department of Mechanical Science and Engineering, University of Illinois, Urbana-Champaign, Urbana, IL 61801, USA.; ^6^ Department of Bioengineering, University of Illinois, Urbana-Champaign, Urbana, IL 61801, USA.; ^7^ Materials Research Laboratory, University of Illinois, Urbana-Champaign, Urbana, IL 61801, USA.; ^8^ Beckman Institute for Advanced Science and Technology, University of Illinois, Urbana-Champaign, Urbana, IL 61801, USA.; ^9^ Nick Holonyak Micro and Nanotechnology Laboratory, University of Illinois, Urbana-Champaign, Urbana, IL 61801, USA.

## Abstract

Soft electronics, known for their bendable, stretchable, and flexible properties, are revolutionizing fields such as biomedical sensing, consumer electronics, and robotics. A primary challenge in this domain is achieving low power consumption, often hampered by the limitations of the conventional von Neumann architecture. In response, the development of soft artificial synapses (SASs) has gained substantial attention. These synapses seek to replicate the signal transmission properties of biological synapses, offering an innovative solution to this challenge. This review explores the materials and device architectures integral to SAS fabrication, emphasizing flexibility and stability under mechanical deformation. Various architectures, including floating-gate dielectric, ferroelectric-gate dielectric, and electrolyte-gate dielectric, are analyzed for effective weight control in SASs. The utilization of organic and low-dimensional materials is highlighted, showcasing their plasticity and energy-efficient operation. Furthermore, the paper investigates the integration of functionality into SASs, particularly focusing on devices that autonomously sense external stimuli. Functionalized SASs, capable of recognizing optical, mechanical, chemical, olfactory, and auditory cues, demonstrate promising applications in computing and sensing. A detailed examination of photo-functionalized, tactile-functionalized, and chemoreception-functionalized SASs reveals their potential in image recognition, tactile sensing, and chemosensory applications, respectively. This study highlights that SASs and functionalized SAS devices hold transformative potential for bioelectronics and sensing for soft-robotics applications; however, further research is necessary to address scalability, long-time stability, and utilizing functionalized SASs for prosthetics and in vivo applications through clinical adoption. By providing a comprehensive overview, this paper contributes to the understanding of SASs, bridging research gaps and paving the way toward transformative developments in soft electronics, biomimicking and biointegrated synapse devices, and integrated systems.

## Introduction

Soft electronics represent a transformative class of electronics distinguished by their ability to bend, stretch, and flex while maintaining their functionality. Unlike traditional rigid electronics, soft electronics can conform to irregular surfaces and tolerate external mechanical deformation, revolutionizing the biomedical sensing, consumer electronics, and robotics fields [1–6]. In the realm of wearable biomedical sensors, a prominent application of soft electronics, there is a substantial advancement in the noninvasive and continuous monitoring of vital signs, including heart rate, blood pressure, chemical compositions, and body temperature. Beyond sensing, soft electronics are integral in prosthetics and tissue engineering, ensuring seamless interfaces with artificial limbs and organs, thus elevating comfort and enhancing functionality [7–10]. Despite their transformative potential, soft electronics encounter various challenges, with one substantial issue being low power consumption. This is a pivotal factor for their practical application and widespread adoption. The predominant barrier in this regard is the traditional von Neumann architecture, which impedes the realization of high energy efficiency in soft electronics due to its segregation of processing and memory elements [11]. Additionally, the inherent rigidity, limited integration with biosystems, and negative environmental impacts often hold back the use of traditional computing schemes for mimicking bioneural parallel processing with softness. Addressing these limitations necessitates the exploration and implementation of a new architecture in soft electronics.

Addressing the inherent challenges in soft electronics, the development of soft artificial synapses (SASs) has emerged as a promising solution, drawing considerable attention in recent research [12–15]. Mimicking the biological synapse, SASs aim to replicate the signal transmission properties of biological systems with low energy consumption [16]. In a biological–neural system, synapses serve as fundamental connections transmitting electrical or chemical signals between neurons and cells [17–19]. This process involves the release of neurotransmitters from the presynaptic terminal, traversing the synaptic cleft, and reaching the receptors on the postsynaptic terminal, governed by synaptic weight control mechanisms. The regulation of the charge and potential of the postsynaptic terminal leads to short-term plasticity and long-term plasticity. Short-term plasticity enables rapid neurophysiological computation of vital information in brain networks, whereas long-term plasticity underpins the formation of lasting memories [20].

Just as the biological synapse processes information using synaptic plasticity, SASs modulate the strength of a connection (synaptic weight) between presynaptic and postsynaptic terminals. This modulation is achieved through a pulse-shaped weight control voltage, resulting in the generation of an excitatory postsynaptic current (EPSC) or an inhibitory postsynaptic current (PSC) [21]. The duration for which the synaptic weight is retained determines short-term and long-term plasticity [22]. Leveraging these electrical behaviors, SASs have been instrumental in constructing brain-like neural network systems, substantially enhancing information processing capabilities [16,23,24]. Furthermore, the soft nature of SASs enables the preservation of synaptic properties even under mechanical deformation. For instance, Fu et al. [25] demonstrated a SAS with a bending radius of nearly 25 mm, capable of enduring a thousand bending cycles. Lee et al. [26] showed that using fluorinated tetrathiophene–diketopyrrolopyrrole-based polymer nanowires with an ion-gel dielectric could demonstrate a 100% straining limit for over 50 training cycles. These breakthroughs highlight the potential of SASs in creating effective interfaces with artificial limbs and organs, a critical advancement in enhancing comfort and functionality within the realm of soft electronics.

**Fig. 1.**
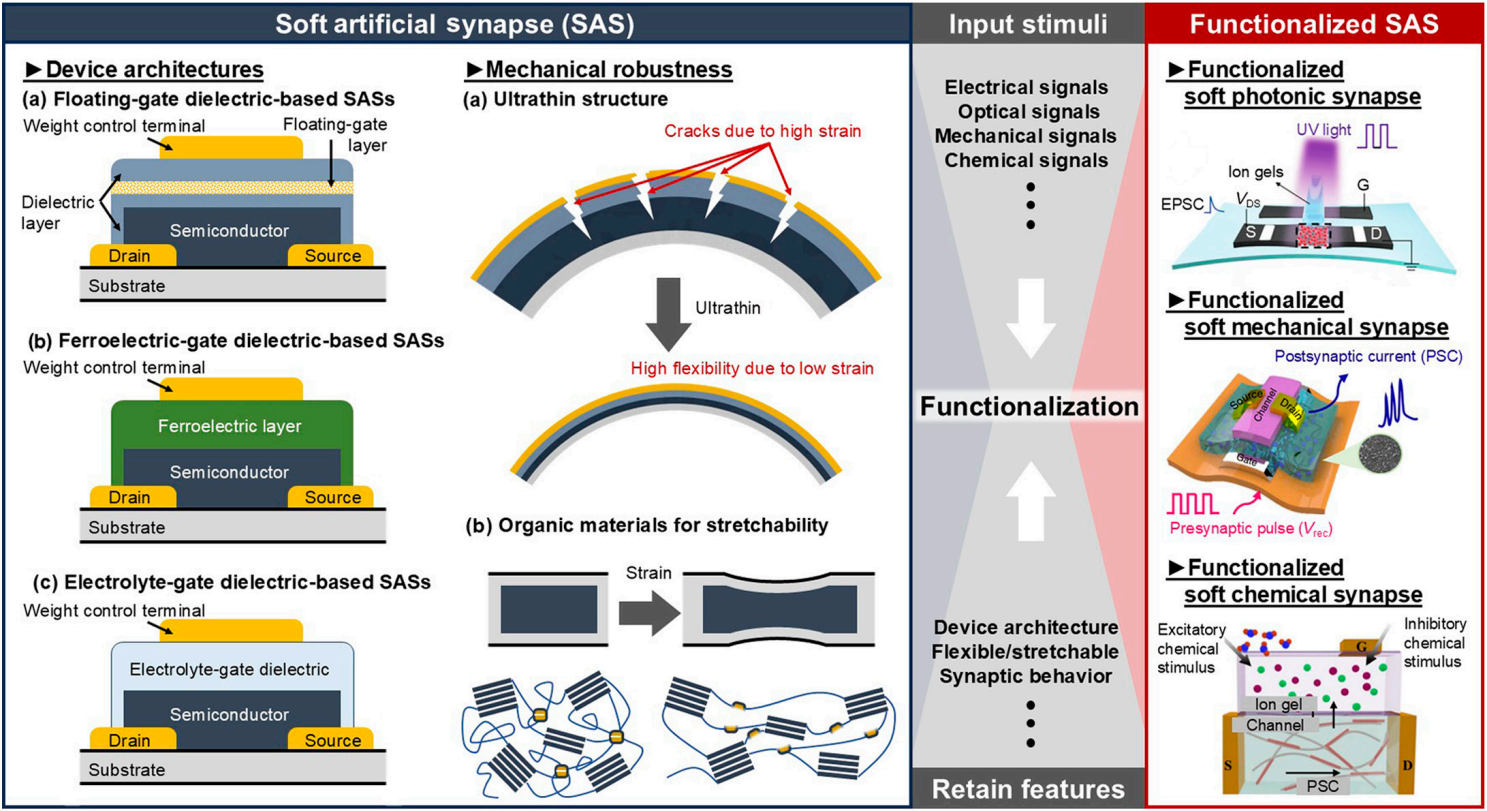
Device architecture realization for soft artificial synapse (SAS) and functionalized SAS. Three basic SAS architectures and material choices for SASs mainly focus on the robustness under strain (left panel). Three functionalized SAS structures based on various input stimuli (right panel) include functionalized soft photonic synapse [27], functionalized soft mechanical synapse [84], and functionalized soft chemical synapse [73]. UV, ultraviolet; EPSC, excitatory postsynaptic current; S, source; D, drain, G, gate.

To construct SASs, various device architectures have been explored, including floating-gate dielectric, ferroelectric-gate dielectric, and electrolyte-gate dielectric, as illustrated in Fig. 1. A critical aspect in the fabrication process is the selection of weight control dielectric and semiconductor materials. These materials are chosen for their ability to maintain consistent synaptic properties, even under mechanical strains such as bending or stretching. This consideration ensures the resilience and reliability of SASs in diverse applications.

In recent advancements, substantial emphasis has been placed on the functionalization of SASs. Functionalized SASs are specialized devices capable of directly sensing and responding to specific external stimuli. These stimuli include optical, mechanical, chemical, olfactory, and auditory cues. The primary function of these devices is to regulate synaptic weight in response to the detected stimuli, thus enhancing the sensory capabilities of SASs [27–32]. This functionalization has opened the door to high-accuracy sensing applications when SASs are optimally designed. For instance, efficient image perception and recognition have already been achieved with functionalized photonic synapses, where the intensity of ultraviolet (UV) light is used to control synaptic plasticity [33,34]. The successful implementation of these functionalized SASs in such capacities showcases the immense potential of SASs in creating more durable, efficient, and environmentally robust soft electronic devices. Understanding the nuances of SASs and their possible applications is vital in advancing the field of soft electronics, potentially revolutionizing the way that we interact with technology.

This review paper provides an in-depth analysis of the materials and architectures essential for developing SASs for advanced applications in soft electronics. It goes beyond the conventional scope by emphasizing functionalized SASs, which demonstrate capabilities for performing synaptic computation at very low energy (approximately tens of picojoules) in response to diverse external stimuli such as optical illumination, mechanical strain, and chemical particles. This dual capability in computing and direct sensing applications, eliminating the need for a dedicated transducer part, highlights their potential to scale down and reduce the complexity of circuit design. Moreover, functionalized SASs show strong potential for clinical application in nerve interfacing. By providing a comprehensive synthesis of the current state and future directions of SAS research, this paper contributes a seminal perspective to the field, aiming to bridge current research gaps and underpin future transformative developments in flexible and responsive electronic devices.

## Materials for SASs

**Fig. 2.**
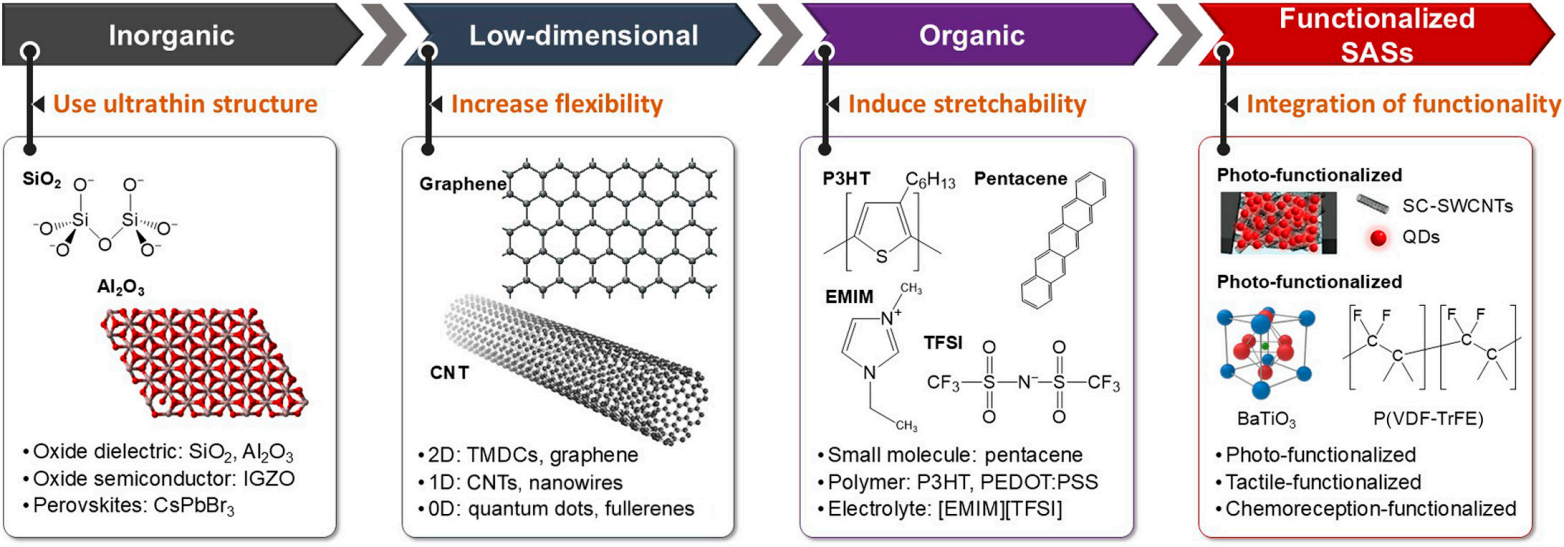
Development of materials for SAS and functionalized SAS realization. Inorganic materials offer limited flexibility, while ultrathin layers and low-dimensional materials improve flexibility. Organic materials provide excellent stretchability due to their polymer structure. The application of stretchable and bendable materials that respond well to external cues like photons, chemical ions or particles, and mechanical strain results in the realization of a functionalized SAS device [27]. IGZO, indium gallium zinc oxide; CNT, carbon nanotube; 2D, 2-dimensional; 1D, 1-dimensional; 0D, 0-dimensional; P3HT, poly(3-hexylthiophene); EMIM, 1-ethyl-3-methylimidazolium; TFSI, bis(trifluoromethanesulfonyl)imide; PEDOT, poly(3,4-ethylenedioxythiophene); PSS, polystyrene sulfonate; SC-SWCNTs, semiconducting single-wall carbon nanotubes; QDs, quantum dots; P(VDF-TrFE), polyvinylidene fluoride-trifluoroethylene; TMDCs, transition metal dichalcogenides.

The selection of materials in the fabrication of SASs is important because it substantially affects the stable operation of artificial synapses under mechanical deformation. The fabrication of SASs using inorganic materials with micrometer-scale thickness exhibits stable behavior to environmental and chemical substances [35–42]. However, the inherent inflexibility, brittleness, and rigidity of inorganic materials pose considerable limitations, especially in the context of applications within soft electronics and wearable technologies. In an effort to overcome these material-specific challenges, researchers have shifted their focus toward organic and low-dimensional materials, as shown in Fig. 2. These materials have emerged as promising alternatives due to their inherent soft behavior and convenient manufacturing processes. This shift marks an important stride in the field, as organic and low-dimensional materials open up new avenues for the fabrication of SAS devices. Their adoption not only addresses the limitations posed by inorganic materials but also aligns with the overarching goals of developing more adaptable, resilient, and user-friendly soft electronic devices.

The employment of low-dimensional materials, such as transition metal dichalcogenide monolayers, graphene, carbon nanotubes (CNTs) [43], and black phosphorene [44], plays a pivotal role in enhancing the flexibility of SASs. The ultrathin structure and unique physical properties of these materials contribute to superior flexibility, a key attribute for SAS applications [2,3]. Furthermore, in the pursuit of creating stretchable electronics, organic materials have emerged as a preferred choice. Their stretchability is primarily attributed to the van der Waals bonding between molecules [1]. Conjugated polymers, such as poly(3,4-ethylenedioxythiophene):polystyrene sulfonate (PEDOT:PSS), polyvinylidene fluoride-trifluoroethylene (P(VDF-TrFE)), polyvinylidene fluoride-hexafluoropropylene, and organic small molecules, including poly(3-hexylthiophene) (P3HT), pentacene and dinaphtho[2,3-*b*:2′,3′-*f*]thieno[3,2-b]thiophene (DNTT), are well investigated for their efficiency and mechanical flexibility in SAS device realization. Furthermore, the dielectrics and semiconductors used in SASs can further be chemically modified to include specific functional groups or composites, paving the way for fabricating functionalized SASs [27,45]. The detailed analysis of organic SASs is prevalent in literature.

## Device Architectures in SASs

The ability to achieve effective weight control in SASs is crucially dependent on the modulation of channel conductance. To facilitate this process, various device architectures have been developed and implemented. The representative architectures for SASs can be classified into floating-gate dielectric-based SASs (FG-SASs), ferroelectric-gate dielectric-based SASs (FeG-SASs), and electrolyte-gate dielectric-based SASs (EG-SASs). When a weight control voltage is applied to each weight control terminal, the dielectric materials endeavor to retain charges in the channel layer, subsequently altering the resistance of the channel and carrier mobility [46]. The enduring nature of the modified state, achieved through the application of electrical pulses or pulse trains, enables SASs to obtain essential synaptic properties such as synaptic plasticity [20–22,47]. These features are essential in a variety of architectures, and the use of different architectures has the potential to be suitably applied to simulate complex nervous systems, contributing to the mimicking of the biological nervous system.

### Floating-gate dielectric-based SASs

**Fig. 3.**
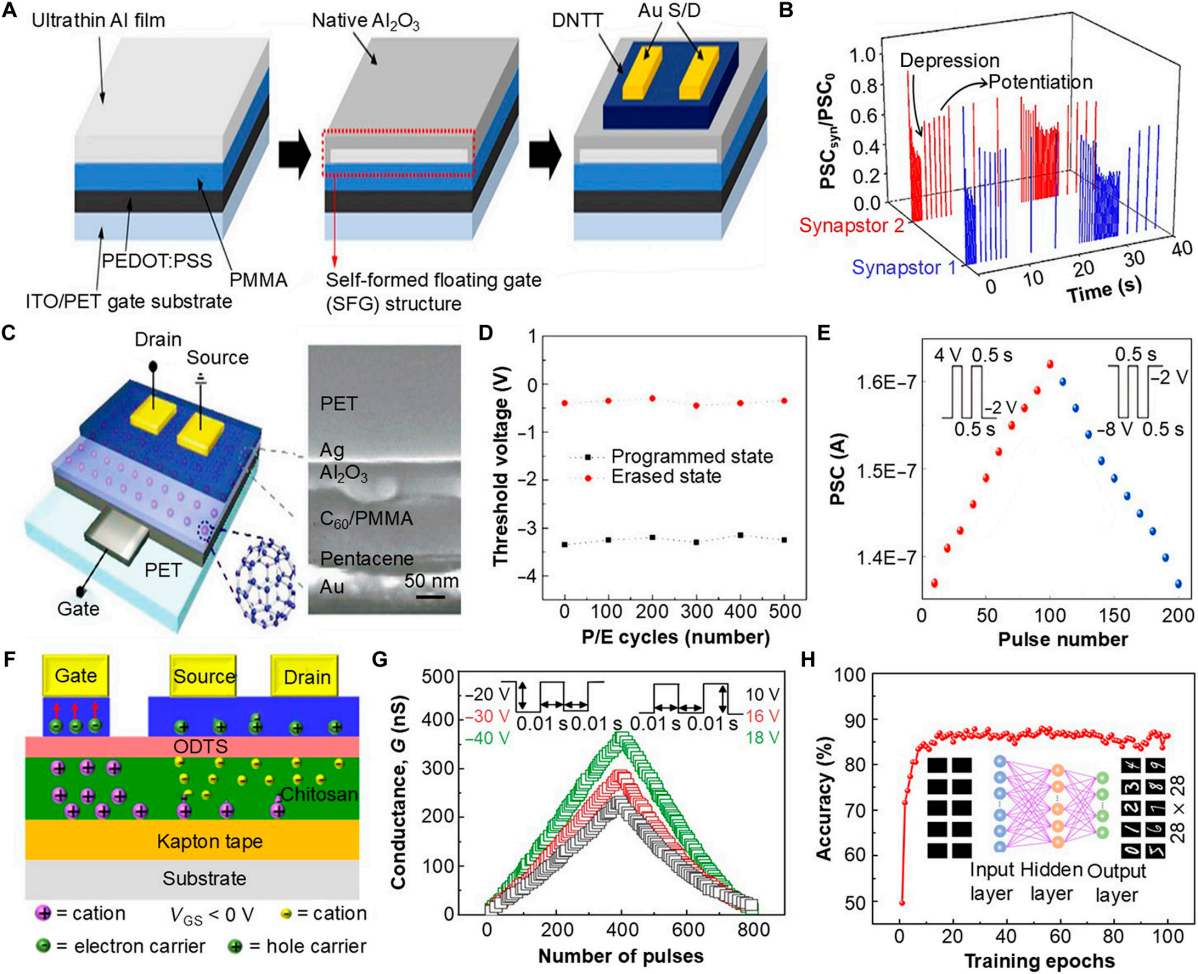
Floating-gate soft artificial synapses (FG-SAS). (A) Depiction of an FG-SAS utilizing the self-form floating-gate (SFG) technique. The Al floating gate helps in charge trapping and conductance modulation of the DNTT semiconductor channel; indium tin oxide (ITO)/PEDOT:PSS/PMMA acts as the common gate electrode/dielectric over the polyethylene terephthalate (PET) substrate. (B) The recorded synaptic currents (*I*_syn_) obtained from selected devices, after being normalized by the initial value *I*_0_ [51]. (C) Left: Schematic illustration of a 3-dimensional, bottom-gate, top-contact SAS transistor. Right: Cross-sectional side-view scanning electron microscopy (SEM) image of the device. (D) Compressive bending cycling test of the SAS. (E) Repetitive synaptic device conductance modulation for potential and depression operation by applying presynaptic positive and negative pulses, respectively. *V*_DS_ was kept at −3 V. Right: Enlarged illustration of one waveform [47]. (F) Diagram illustrating flexible floating-gate electric-double-layer (EDL) synaptic transistors constructed using a pentacene-based channel and a floating layer. (G) Illustrations of the achieved long-term potentiation (LTP) and long-term depression (LTD) conductance for different pulse amplitudes. The gate voltage and drain voltage pulse duration was kept at 20 ms. (H) Graphical representation of the 3-layer perceptron-based artificial neural network’s performance relative to the count of training epochs for the test set of handwritten digit images (0 to 9). Inset: Neural network designed for identifying patterns in 28 × 28 grayscale images, featuring input, hidden, and output layers [53]. PSC, postsynaptic current; P/E, Programming/Erase.

The architecture of FG-SASs is defined by the inclusion of a floating-gate layer within the dielectric layer, a design that endows these devices with memory properties [47–50]. Table 1 presents a comprehensive summary of FG-SASs. To fabricate the architecture, 3 steps are required: (a) deposition of a tunneling dielectric layer, (b) deposition of a floating-gate layer, and (c) deposition of a dielectric layer to embed the floating-gate layer, enabling nonvolatile memory characteristics by trapping charges in the floating-gate layer. To improve the processability of SAS manufacturing, Kim et al. [51] reported a flexible synaptic transistor with a vacuum-deposited charge-trapping nanosheet floating-gate layer. An Al nanosheet (3 nm), exposed to the ambient air, formed native oxide working as the tunneling dielectric layer (Fig. 3A). The self-formed Al floating-gate architecture acts as a charge-trapping layer and induces synaptic plasticity. The trapped charge layer, being placed between the top weight control terminal and the semiconductor, could effectively shield the vertical electric field from the weight control terminal to the semiconductor. As such, the amount of charge induced in the semiconductor layer is controlled by applying pulse trains to the weight control terminals, as shown in Fig. 3B. This process effectively facilitates synaptic weight change and thus is a promising platform for simplifying the manufacturing process of SASs. Furthermore, the proposed device demonstrates mechanical flexibility, maintaining its functional integrity under moderate stress conditions, which further underscores the viability of FG-SASs in various applications.

**Table 1.** Summary of FG-SASs

Semiconductor material	Dielectric material	Bending radius/strain	Bending cycles	Stretching strain	Stretching cycles	Application	Functionalization	Ref.
BP	BP/Al_2_O_3_/BP/HfO_2_	10 (mm)	5,000 (0.63%)	-	-	Neuromorphic application	-	[83]
SC-CNT	SiO*_x_*/Au/SiO*_x_*	-	-	-	-	Pattern recognition	-	[52]
Pentacene	C_60_/PMMA/Al_2_O_3_	10 (mm)	500	-	-	Neuromorphic computing	-	[47]
Pentacene	Pentacene/ODTS/chitosan/Kapton tape	90° bending	-	-	-	Neuromorphic computing	-	[53]
DNTT	PEDOT:PSS/PMMA/Al nanosheet	7.5 (mm)	-	-	-	Neuromorphic systems	-	[51]
SC-CNT	Al_2_O_3_/HfO_2_/AlO*_x_* NPs	-	2,000 (0.4%)	-	-	Artificial sensory memory	PF-SAS	[107]
MoS_2_	Al_2_O_3_/ZrO_2_/Al_2_O_3_	10 (mm)	-	-	-	Wearable neuromorphic computing	PF-SAS	[108]
MoSSe	Al_2_O_3_/BP QDs/Al_2_O_3_	5 (mm)	1,000	-	-	Wearable neuromorphic computing	PF-SAS	[82]

FG-SASs, floating-gate dielectric-based soft artificial synapses; BP, black phosphorus; SC-CNT, semiconducting carbon nanotube; PMMA, poly(methyl methacrylate); ODTS, octadecyl trichlorosilane; DNTT, dinaphtho[2,3-*b*:2′,3′-*f*]thieno[3,2-b]thiophene; NPs, nanoparticles; PF-SAS, photo-functionalized soft artificial synapse

Further exploring improving the processability, Ren et al. [47] introduced a novel approach by incorporating a fullerene (C_60_)/poly(methyl methacrylate) hybrid layer, replacing the conventional deposition of the tunneling dielectric and floating-gate layer as shown in Fig. 3C. In the hybrid layer, trapping sites of fullerene are doped in poly(methyl methacrylate) by a facile solution process, and the polarity of charge trapping could be controlled with a low weight control voltage (<5 V). The electrical properties of the device did not show a substantial change in the cyclic bending measurement (bent with a radius of 10 mm), indicating mechanical stability (Fig. 3D). Furthermore, this low-voltage operation with linear long-term potentiation (LTP)/long-term depression (LTD) is shown in Fig. 3E. Excellent linear weight update, stability of the threshold voltage over repeated cycles of operation, high transconductance, and repetitive ion-concentration-dependent switching properties are important characteristics to be sought after for effective neuromorphic computing (i.e., brain-inspired computing) applications [52]. In 2022, Zheng et al. [53] reported a planar flexible FG-SAS that effectively demonstrated a symmetric weight update with low variation over repeated operation. In addition, the proposed device reported more than 800 conducting states and switching endurance over 100 cycles. Figure 3F provides an illustration of the proposed electrolyte-based FG-SASs with pentacene. Here, the chitosan electrolyte layer with an octadecyl trichlorosilane blocking layer induces charge trapping. The floating-weight control terminal layer can change the energy band substantially and facilitate charge transport via thermal emission or quantum tunneling. As a result, the charge from the channel can migrate to the floating-weight control terminal, and due to the robust blocking nature of the device, these charges stay trapped within it. These trap charges could help provide a nonvolatile or static memory behavior [52,54–56]. The proposed device shows a symmetric LTP/LTD behavior (Fig. 3G). In addition, the work shows the capability to perform data analysis by building an artificial neural network (ANN) of input (784 neurons), hidden (100 neurons), and output layers (10 neurons) (Fig. 3H). When a 28 × 28 conductance image is given (inset left) to the proposed neural network model, it can predict the digits with a nearly 87% accuracy. The mapped images of the Modified National Institute of Standards and Technology (MNIST) database handwritten digits from the array show effective recognition of the digits (inset right) after 100 training epochs. The proposed device layout could be effective for futuristic hardware-processed neuromorphic computing. The linear synaptic weight modulation makes it a competent option for next-generation computing units.

### Ferroelectric-gate dielectric-based SASs

FeG-SASs are well-known for delivering nondestructive memory operation [49,57]. In general, ferroelectric materials are used for switching between 2 remnant polarization states for the modulation of synaptic weight [58–61]. Commonly used inorganic ferroelectric materials in the literature include BiFeO_3_, BaTiO_3_, HfZrO*_x_*, Pb(Zr, Ti)O_3_ (PZT), and Hf_0.5_Zr_0.5_O_2_ [62]. A FeG-SAS was proposed by Li et al. [61] that uses a HfZrO*_x_* dielectric layer with a TiN layer and demonstrated an excellent MNIST handwritten digit recognition accuracy and a high linear synaptic behavior under different pulse conditions. However, the inherent inflexibility of the materials and the high-temperature process for achieving higher crystallinity make it difficult to fabricate the SAS on soft substrates. In response to the challenges posed by inorganic materials in FeG-SASs, researchers have shifted focus to organic ferroelectrics, which offer inherent flexibility and can be fabricated at lower temperatures [63]. As a result, flexible organic semiconductor materials combined with organic ferroelectric polymers ensure mechanical flexibility and ferroelectric switching. For instance, devices made of pentacene combined with a P(VDF-TrFE) ferroelectric dielectric exhibit a bending radius as low as 0.05 mm and withstand up to 100,000 bending cycles [64]. An overview of FeG-SAS electronics can be found in Table 2.

**Table 2.** Summary of FeG-SASs

Semiconductor material	Dielectric material	Bending radius/strain	Bending cycles	Stretching strain	Stretching cycles	Application	Functionalization	Ref.
Pentacene	P(VDF-TrFE)	0.05 (mm)/0.48%	-	-	-	Wearable intelligent electronics	TF-SAS	[64]
Pentacene	Barium titanate NPs/P(VDF-TrFE)	-	100,000	1.25%	-	Neurorobotics (intelligent electronic skin)	TF-SAS	[84]
P(IID-BT)	P(VDF-TrFE)/P(VP-EDMAEMAES)	0.1 (mm)	-	-	-	Artificial visual-perception system	PF-SAS	[109]

FeG-SASs, ferroelectric-gate dielectric-based soft artificial synapses; TF-SAS, tactile-functionalized soft artificial synapse; P(IID-BT), poly(isoindigo-*co*-bithiophene); P(VP-EDMAEMAES), poly[(1-vinylpyrrolidone)-*co*-(2-ethyldimethylammonioethyl methacrylate ethyl sulfate)]

**Fig. 4.**
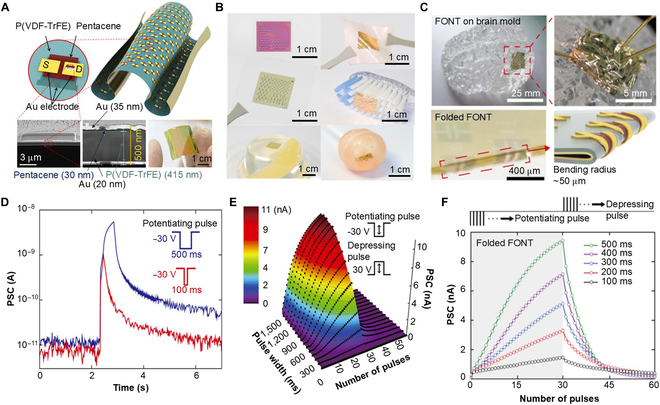
FeG-SASs. (A) Schematic device structure of a FeG-SAS using P(VDF-TrFE) and pentacene. The channel dimensions are specifically measured, with a length of 20 μm and a width of 200 μm. Bottom left: A cross-sectional SEM image of a SAS in its freestanding state. Bottom middle: An enlarged cross-sectional SEM image. The SAS’s overall thickness is approximately 500 nm. Bottom right: Bendable feature of the proposed SASs. (B) Description of SASs that have been transferred and set in a freestanding form onto various substrates, including SiO_2_ and textiles, and in their standalone state, as well as on unconventional bases like a toothbrush, jelly, and candy. (C) Photographs showcasing the SASs adapted to the contours of a brain-shaped polydimethylsiloxane (PDMS) mold (top), along with images of completely folded SASs (bottom), demonstrating a bending radius of approximately 50 μm. (D) PSC responses for different, single presynaptic pulses of −30 V for 100 ms (red line) and 500 ms (blue line). (E) Three-dimensional contour graph depicting the PSC varying with the pulse width (ranging from 100 to 1,500 ms in steps of 100 ms) and the number of pulses, which includes 30 potentiating pulses at −30 V and 30 depressing pulses at +30 V). (F) PSC response of the folded SAS, analyzed based on the pulse width and the number of pulses, which includes 30 potentiating pulses at −30 V and 30 depressing pulses at +30 V [64]. FONT, ferroelectric organic neuromorphic transistor.

In order to apply SASs to various curved surfaces, Jang et al. [64] reported ultrathin (~500-nm-thick) organic FeG-SASs, as shown in Fig. 4A. The proposed FeG-SASs can retain their functionalities even in a freestanding condition, which facilitates their transferability onto diverse substrates of random surfaces and shapes (Fig. 4B). Furthermore, the devices demonstrated remarkable mechanical resilience; they were folded to a bending radius of just 0.05 mm with 0.48% straining and successfully attached to the brain surface, as illustrated in Fig. 4C. To explore the synaptic properties, various weight control voltages are applied to control the amount of polarization in these ferroelectric materials, resulting in the modulation of synaptic weight. Figure 4D shows the PSC behavior under −30 V. A potentiating pulse was applied for 100 and 500 ms. With the increase in the pulse width of the presynaptic pulses, a high PSC was observed. In addition, the authors also showed the relationship between PSC according to the modulation of pulse numbers and widths (Fig. 4E). Moreover, the synaptic property under folded conditions was investigated with varied pulse width and numbers. As shown in Fig. 4F, even at the folded condition, both the LTP and LTD behaviors closely mimic the behavior realized with the pristine, unstrained proposed FeG-SAS architecture. This exceptional folding limit, combined with synaptic retentivity, positions these organic FeG-SASs as a highly competent option for next-generation wearable electronics applications.

### Electrolyte-gate dielectric-based SASs

In EG-SASs, an electrolyte dielectric layer is used, which can enhance low-voltage operation for logic design and ANN application. Electrochemical and mechanical flexibility are 2 common features usually considered when choosing the correct electrolytic dielectric material. Inorganic materials, such as sodium alginate and lithium silicate oxide, were used as electrolyte-gate architectures [65–71]. However, the inherent inflexibility of these materials has somewhat limited their consideration for EG-SAS realization. Organic materials are often a first-door choice for highly stretchable device structures. Commonly used soft organic electrolytic dielectric materials include poly[2-methoxy-5-(20-ethylhexyloxy)-*p*-phenylene vinylene]/RbAg_4_I_5_ [72], PEDOT:PSS [29,73,74], and ion gels [23,25,69,75]. In addition, material combinations, such as composites of P3HT core and polyethylene oxide sheath and cellulose nanopaper with ionic liquid, are reported to show ultralow energy consumption (1.23 fJ), flexibility, and biodegradable operation [76,77]. Integrating highly stretchable semiconductors with flexible electrolytes ensures synaptic weight adjustment with minimal energy input. Table 3 summarizes the common EG-SAS architectures.

**Table 3.** Summary of EG-SASs

Semiconductor material	Dielectric material	Bending radius/strain	Bending cycles	Stretching strain	Stretching cycles	Application	Functionalization	Ref.
P3HT NFs	PVDF-HFP/[EMIM][TFSI]	20 (mm)	1,000	-	-	Artificial limbs, robotics, and bionics	-	[89]
P3HT NFs	PAN/[Li][TFSI]	-	-	60%	300 (30%)	Soft neuromorphic systems	-	[85]
P3HT NFs/PDMS	PVDF-HFP/[EMIM][TFSI]	-	-	30%	-	Tactile neural sensors	-	[15]
P3HT NFs/PDMS	PVDF-HFP/[EMIM][TFSI]	-	-	50%	1,000 (50%)	Tactile neural sensors	-	[81]
P3HT NFs/PDMS	PVDF-HFP/[EMIM][TFSI]	-	-	30%	-	Neurocognitive skin	-	[32]
SC-SWCNT	PVA/SiO_2_	-	-	50%	1,000 (30%)	Multimodal neuromorphic electronic skin	-	[94]
SC-SWCNT, CdSe/ZnS QDs	PS-PMMA-PS/[EMIM][TFSI]	-	-	20%	-	Neural bionic eye (pattern recognition)	PF-SAS	[27]
PbS QDs/PDPP-DTT	PVA/PVP	1 (mm)	-	-	-	Artificial night vision system	PF-SAS	[110]
SnO_2_ NP	PMMA/LiClO_4_	8 (mm)	10,000	-	-	Artificial sensory nerves	PF-SAS	[50]
PEDOT:PSS	NaCl ion gel	7.5 (mm)	-	-	-	Biohybrid integration into tissues	CF-SAS	[29]
PEDOT:PSS	PEGDA/[EMIM][TFSI]	-	-	-	-	Biomimetic chemosensory	CF-SAS	[73]
DPP-DTT	PDMS	7 (mm)	-	-	-	Pattern recognition	PF-SAS	[93]
IGZO	Chitosan/graphene oxide	10 (mm)	1,000	-	-	Photoexcited corneal nociceptor	PF-SAS	[111]

EG-SASs, electrolyte-gate dielectric-based soft artificial synapses; NFs, nanofibrils; PVDF-HFP, poly(vinylidene fluoride-*co*-hexafluoropropylene); PAN, polyacrylonitrile; PVA, polyvinyl alcohol; PS, polystyrene; PVP, polyvinylpyrrolidone; CF-SAS, chemoreception-functionalized soft artificial synapse; PEGDA, polyethylene glycol diacrylate; PDPP-DTT, poly(diketopyrrolopyrrole–dithienothiophene); DPP-DTT, diketopyrrolopyrrole-dithienylthieno[3,2-*b*]thiophene

**Fig. 5.**
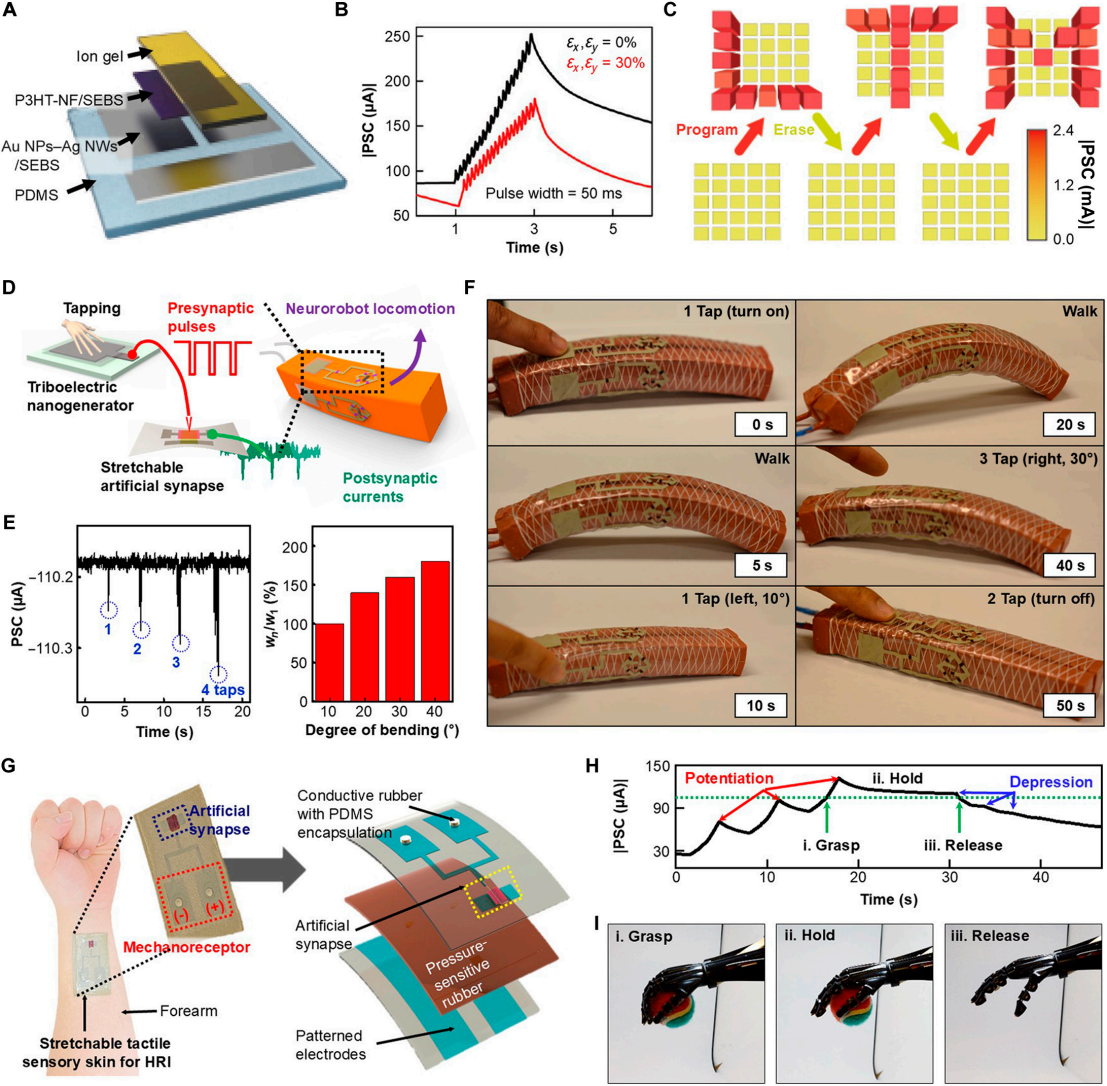
EG-SASs. (A) Schematic view of a biaxially stretchable elastomeric EG-SAS. (B) Synaptic EPSC characteristics of a synaptic transistor without (black) and with (red) biaxial mechanical strain. (C) Implementation of a program and erase capability of pixelated letters “L”, “T”, and “M” through synaptic potentiation and depression behavior in a 5 × 5 transistor array. For programming and erasing, 60 successive presynaptic pulses of −4 and 6 V of 500-ms pulse width and 2-Hz pulse frequency are applied, respectively [32]. (D) Illustration of a neurologically integrated soft robot, depicting its locomotion operation configuration. (E) Left: The variation in EPSC with varying numbers of taps. Right: Graph showing the bending angle of the programmed soft robot as it correlates with the short-term weight change (represented as *W_n_*/*W*_1_). (F) Image sequence depicting the motion of a soft neurorobot, adjusting adaptively in response to the signals from robotic memory [81]. (G) Left: Optical image showing the flexible neurological electronic skin applied for human–robot interaction (HRI), attached to the forearm. Right: Detailed exploded view of the flexible neurological electronic skin HRIs. (H) EPSC during various robotic hand motions, including grasping, holding, and releasing an object, over a duration of 46 s. (I) Sequence of images depicting the robotic hand’s grasp, hold, and release operations, guided by the synaptic currents from a fully flexible, entirely organic synaptic transistor [15]. NWs, nanowires; SEBS, styrene-ethylene-butylene-styrene.

The charge transfer in an electrolyte dielectric layer starts with a relatively low weight control voltage. As a result, EG-SASs are crucial for ultralow power synaptic applications [78–80]. Shim et al. [32] reported a biaxially stretchable elastomeric synaptic transistor (Fig. 5A) that uses a P3HT/styrene-ethylene-butylene-styrene channel layer with an ion-gel electrolytic dielectric. The device could be stretched up to 30% biaxial strain, twisted, and poked. The EPSC curve for the proposed device with and without strain simultaneously demonstrates both short-term memory and long-term memory characteristics (Fig. 5B). A 5 × 5 array of elastomeric transistors was also illustrated with dynamic programming and erasing capability. As shown in Fig. 5C, applying a pulse train of 60 pulses with a 2-Hz frequency, a 500-ms pulse width, and −4-V presynaptic voltage results in a rise of the EPSC value, which can be encoded as the “program” stage. When a 6-V presynaptic voltage is applied with similar properties, the device demonstrates a depression trend, which was encoded as “erase”. It was observed that the proposed device could be dynamically programmed and erased with 60-pulse trains. The proposed method was then used to demonstrate the memorizing and deleting option for the pixelated letters “L”, “T”, and “M”.

Continuing from the exploration of EG-SASs, the field advances with the introduction of stretchable, rubbery synaptic transistors, marking an important stride in applications like locomotive motion and soft robotics. This innovative approach includes the development of a fully elastomeric synaptic transistor, which integrates P3HT as the semiconductor and PVDF-HFP/[EMIM][TFSI] (PVDF-HFP = poly(vinylidene fluoride-*co*-hexafluoropropylene); EMIM = 1-ethyl-3-methylimidazolium; TFSI = bis(trifluoromethanesulfonyl)imide) as the electrolytic dielectric over a polydimethylsiloxane (PDMS) substrate, further enhanced by an external mechanoreceptive triboelectric nanogenerator (TENG) system, as illustrated in Fig. 5D [81]. This system adeptly converts external mechanical stimuli, such as tapping, into electrical presynaptic pulses for the transistor. When the transistor is exposed to pulse trains comprising 20 successive pulses, each 50 ms in duration, at a frequency of 10 Hz and an amplitude of −3 V, it exhibits both short-term and long-term memory capabilities. The proposed device requires energy per synaptic event as low as 1.23 fJ.

Notably, as demonstrated in Fig. 5E, the EPSC values gradually increase with the frequency of tapping on the TENG, set at a 50% duty cycle and 5 Hz. Furthermore, a higher short-term weight ratio (*W_n_*/*W*_1_) leads to a greater degree of bending, indicative of the potential for adaptive memory in robotic applications. The research innovatively integrates synaptic transistors into the realm of soft robotics, leading to the conception of neurologically integrated, adaptive robots. The design incorporates a unidirectional electrical output, achieved by embedding a soft, full-wave bridge rectifier. Strategically positioned mechanoreceptive skins on different sides of the neurorobot enable precise control over its movements. As shown in Fig. 5F, the number of taps on either the left or right skin determines the robot’s turning direction and angle, demonstrating a sophisticated level of control. The top skin serves the crucial function of initiating or halting the robot’s movement through a system of simple taps.

A novel application of EG-SASs is in the development of a neurological electronic skin, designed for attachment to the human forearm [15]. This skin utilizes synaptic transistor functionalities to communicate with a robotic hand using tactile signals encoded in basic Morse code, as detailed in Fig. 5G. The architecture of this device includes simple mechanoreceptors made of conductive rubber, encased in PDMS, forming part of an artificial synapse. The rubbery nature of the device ensures conformability to human skin and resilience to stretching up to 30%. The mechanoreceptors, when activated, send action pulses to the synaptic transistor. The resultant EPSCs are decoded into Morse code, controlling robotic movements such as left, right, up, and down. Figure 5H illustrates the EPSC responses during a sequence of robotic actions—grasping, holding, and releasing an object. The sequence of grasp, hold, and release, integral to the fully rubbery human–robot interaction demonstration, is detailed in Fig. 5I. This tactile stimulus-based human–robot interaction not only integrates pressure-sensitive rubber with synaptic devices but also represents a substantial advancement in the field of neurological function implementation and neurorobotics. Consequently, the proposed device architecture holds great potential for future research in neurological function implementation and neurorobotics.

## Comparative Performance Analysis of the SAS Architectures

The current investigations carried out on the aforementioned 3 broad SAS device architectures implicitly provide some crucial insights into the unique advantages and limitations of specific applications. FG-SASs offer moderate flexibility with bending radii down to 5 mm and enduring up to 1,000 bending cycles [82], can show picojoule energy spent per synaptic operation [83], making them suitable for neuromorphic computing tasks requiring precise synaptic control; however, they lack high stretchability. FeG-SASs exhibit exceptional flexibility, achieving bending radii as low as 0.05 mm with 0.48% strain [64], withstanding up to 100,000 bending cycles [84] and consuming only tens of picojoule per synaptic event [64], which makes them ideal for wearable electronics and neurorobotics that demand devices conform to complex surfaces, although they may have limited stretching strain capacity (up to 1.25%). EG-SASs provide the highest stretchability, accommodating strains up to 60% [85] and maintaining performance over 1,000 cycles at 50% strain [81], and energy per synaptic event as low as 1.23 fJ, rendering them optimal for applications in soft robotics and tactile sensors where substantial mechanical deformation occurs, despite potential challenges in long-term stability and environmental sensitivity. Thus, consideration of the sought-after mechanical and device performance is crucial to properly select the better-suited SAS architecture; FG-SASs meet precision and moderate flexibility, FeG-SASs enable extensive bending flexibility, and EG-SASs provide high stretchability. Based on mechanical softness with low operating energy, SASs provide unique benefits compared to the traditional soft sensing devices used in bioelectronics and soft robotics. Thus, in next-generation soft electronics and bioelectronics, where higher energy consumption and limited mechanical flexibility could restrict the use of traditional soft electronics, SAS devices are expected to play a significant role—providing high mechanical flexibility, low energy operation, and sensing and computing facilities in a single device.

Despite their promising potential, SASs face challenges in the scalability, long-term durability, and performance metrics compared to traditional computing and sensing devices [86]. Addressing these issues requires advancements in direct stimulus applications to gate terminals and the development of novel fabrication methods tailored to SASs. These efforts would simplify device integration, enhance scalability, and enable SASs to large-scale applications, solidifying their role in flexible, low-power electronics. To that end, investigations on skin-inspired soft bioelectronic and self-healing materials further enhance the capability and adaptability of SASs, highlighting their potential to overcome existing limitations and drive innovation in bioelectronics and related fields [87,88].

## Functionalized SASs

In functionalized SAS device architecture, there is no requirement for additional sensors or transducing components to receive external stimuli. These functionalized SAS devices can directly process external inputs—mechanical deformation, chemical molecules, or photonic exposure—to their channel or gate layer and control the synaptic plasticity based on the magnitude of the external stimuli. The unique capability stems from integrating appropriate stimulus-receptive material within the SAS material library, which enables functionalized features. In functionalized SASs used for tactile and e-skin applications, ion-gel dielectrics with EMIM–TFSI engineering are prevalent over polyimide, polyethylene terephthalate, or PDMS substrates [23,89,90]. The semiconductor materials are selected from the library of organic materials—PEDOT:PSS [29,91], P3HT film [92], and DPP-DTT [93]. In addition, low-dimensional materials are also getting attention for channel layers due to their inherent mechanical strength to withstand external stress. The use of ZnO nanowires [28], semiconducting single-wall CNTs (SC-SWCNTs) [94], 2-dimensional MoS_2_ [67], and thin-layer indium tin oxide [68] is well investigated for biohybrid SAS integration into tissues. The following section details the 3 important categories of functionalized SAS devices that use materials from the SAS material library and shows promises to expand the current thinking and prospects of possible futuristic bioelectronics and soft-robotics design.

### Photo-functionalized SASs

**Fig. 6.**
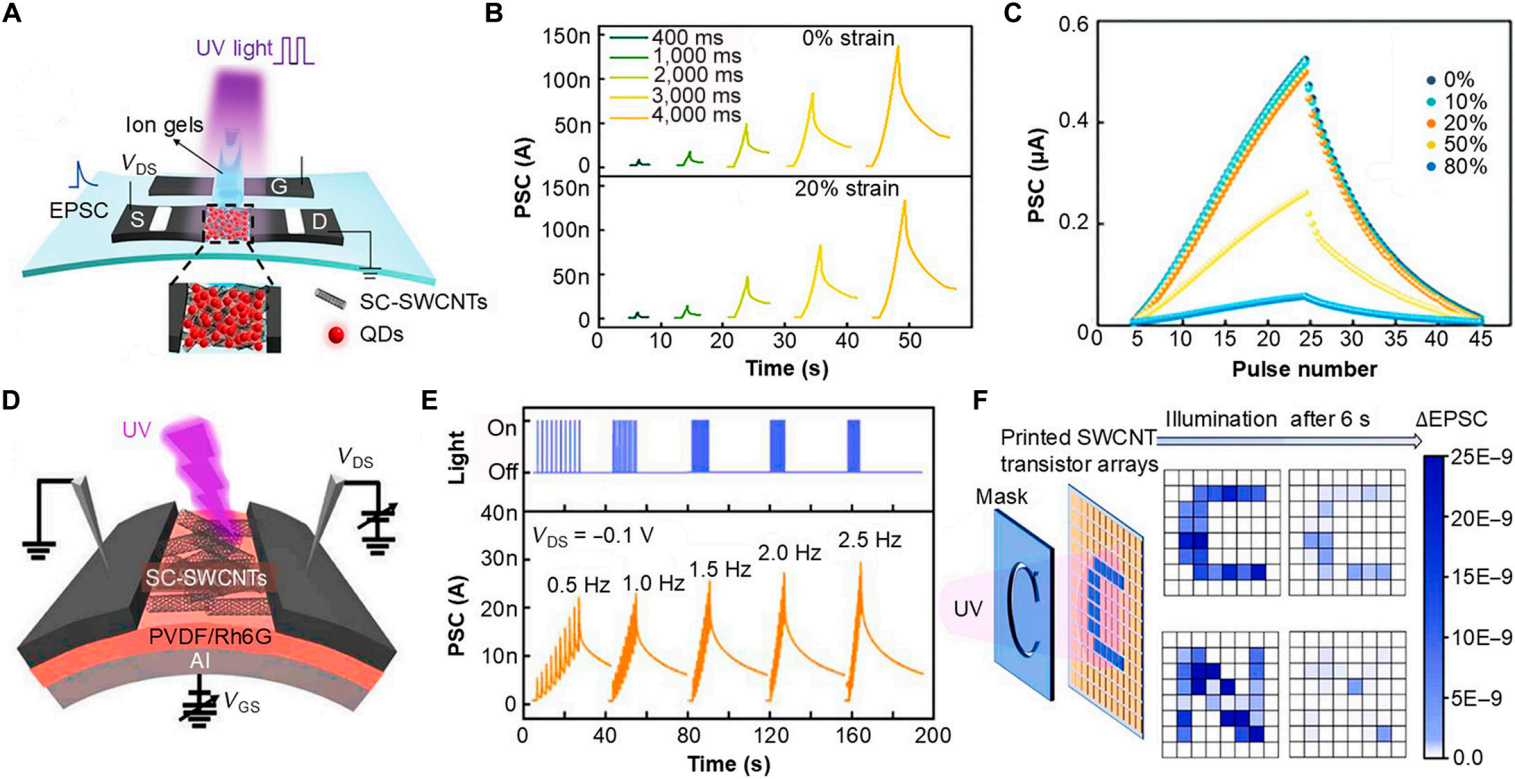
PF-SASs. (A) Schematic illustration of a PF-SAS with an SC-SWCNT channel layer. (B) EPSC observed in the presence of different optical (UV) pulse widths when the postsynaptic terminal voltage is kept at −0.3 V. (C) Variation of LTP/LTD response with UV pulses when the strain is changed from 0% to 80% [27]. (D) Illustration of an SC-SWCNT-based PF-SAS in the presence of optical illumination. (E) EPSC variation when pulse trains of 10 UV light pulses of various frequencies are exposed to the synaptic transistor; the postsynaptic terminal voltage was kept at −0.1 V. (F) Simulated illumination outcomes employing an 8 × 8 pixel grid to depict the letters “C” and “N” while tracking variations in word retention over time [33]. Rh6G, rhodamine 6G.

Photo-functionalized soft artificial synapses (PF-SASs) represent a pioneering development in soft electronics, with the ability to adjust the synaptic weight in response to external light stimuli, eliminating the need for external photodetectors. PF-SASs provide a large bandwidth and a low interconnection energy loss, and are useful for ultrafast signal transmission when attempted for novel ANN architecture [95]. For the fabrication of PF-SASs, Xie et al. [27] introduced SC-SWCNTs modified with CdSe/ZnS quantum dots as the active layer for photonic response (Fig. 6A). The proposed device exhibits ultralow power consumption (15.38 aJ) and can be stretched to 20% without substantially losing its synaptic characteristics under various light (365 nm) pulse widths (Fig. 6B). It was observed that when a UV pulse train (~20 pulses) was applied, the device showed an increased EPSC amplitude. Moreover, the change in the LTP/LTD of the proposed soft synaptic devices at different input pulse numbers under 0% to 80% stretching strain is shown in Fig. 6C.

To enhance processibility, in 2023, Wang et al. [33] reported a CNT-based large-area flexible PF-SAS for image recognition. The roll-to-roll gravure printing technology was used to manufacture a 33 × 34 synaptic transistor array. As shown in Fig. 6D, the device is bendable in nature, and UV light (365 nm) passes through the SC-SWCNTs to obtain the built-in potential in the PVDF-HFP/rhodamine 6G dielectric layer. Figure 6E shows the EPSC characteristics of the proposed device under various frequencies of light pulses. Just as the EPSC of a conventional SAS increases in a higher-frequency presynaptic voltage pulse, the EPSC of the proposed PF-SAS also increases in a high-frequency light pulse. Moreover, based on the synaptic properties, the learning-and-forgetting behavior was obtained by exposing light pulses as a form of the letters “C” and “N” to an 8 × 8 PF-SAS array (Fig. 6F). The device memorized the letters for nearly 6 s and consumed only 0.03 fJ energy/pulse-stimulus, indicating the potential for next-generation SASs.

### Tactile-functionalized SASs

**Fig. 7.**
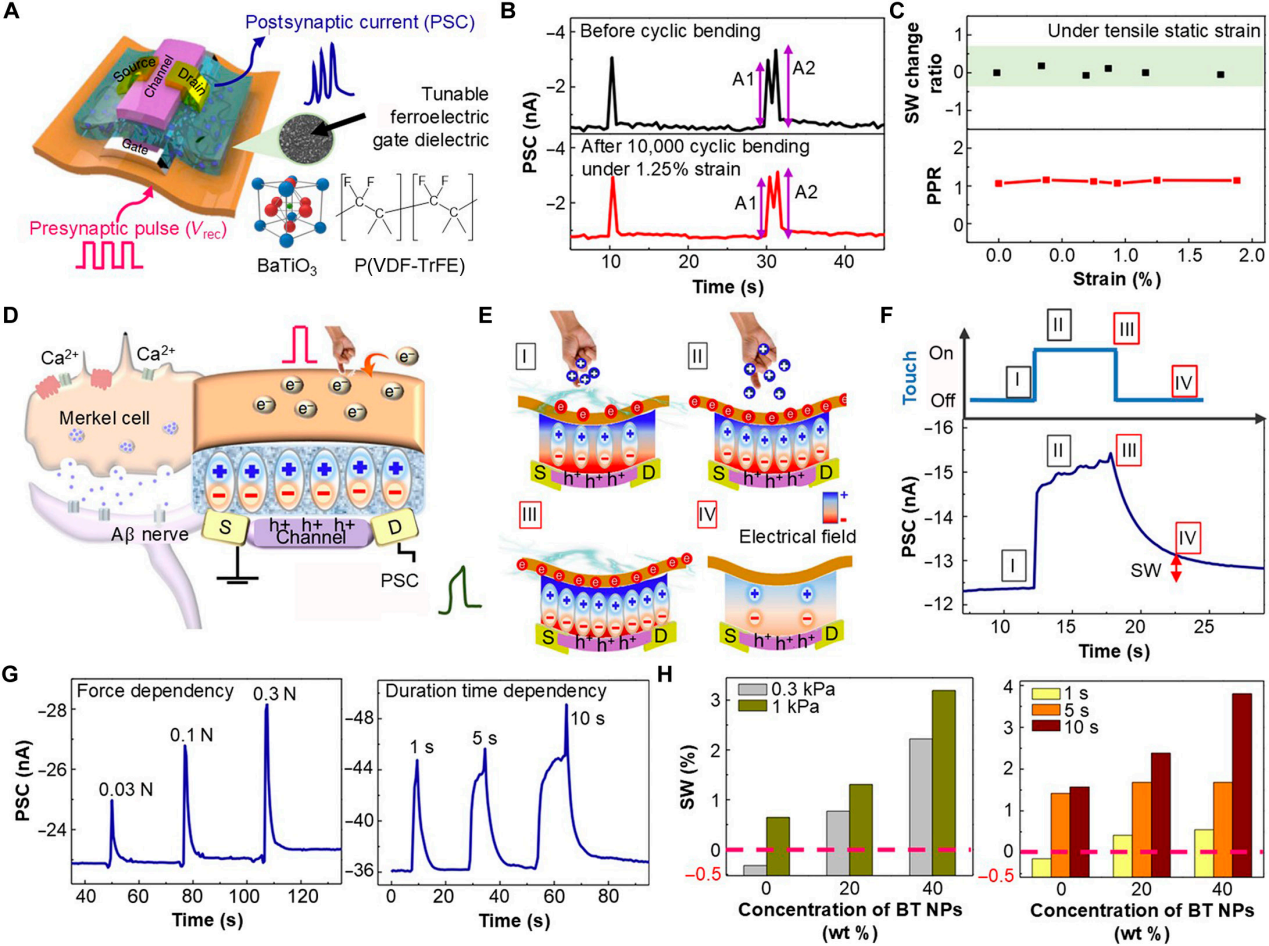
Functionalized soft mechanical synapse (FSMS). (A) Schematic illustration of BaTiO_3_ NPs/P(VDF-TrFE) ferroelectric material dielectric-based FSMS. (B) Variation in PSC before and after a cyclic bending test was deployed, using a pulse train of −10-V presynaptic gate pulses with a 500-ms pulse width and a 500-ms pulse interval. (C) Change in synaptic weight and PPR when 0% to 2% static tensile strain is incorporated. (D) Illustration of a Merkel cell and Merkel cell–neurite complex structure. (E) Schematic depiction of 4 states (I to IV) of dipole generation and charge carrier transfer between the ferroelectric layer and the channel layer in the presence of tactile stimulation. (F) Variations in PSC under a tactile stimulus of around 0.3 kPa and a postsynaptic terminal bias of −3 V for states I to IV. (G) Change in PSC when the tactile pressure (left) and the duration of tactile touch (at ~1 kPa) (right) are altered. (H) Variation in synaptic weight with 2 different pressures (left) and 3 different time intervals of the tactile pulse (right) when the concentration of BaTiO_3_ NPs is kept at 0%, 20%, and 40% [84]. SW, synaptic weight; BT, barium titanate; PPR, paired pulse ratio.

A tactile-functionalized soft artificial synapse (TF-SAS) represents a novel architecture for sensing external mechanical stimuli, particularly in applications like tactile sensing, artificial skin, and soft limbs. Traditional tactile sensing in SASs often relies on external tactile sensors coupled with spike coding circuits and piezoelectric or triboelectric components to capture mechanical stress or deformation. These components then convert the sensed mechanical input into presynaptic pulses for the synaptic device. However, TF-SASs eliminate the need for these dedicated tactile sensor parts, offering a more compact and efficient solution. Recently, Lee et al. [84] proposed an organic, flexible synaptic transistor to be used as a tactile sensory organ. As shown in Fig. 7A, barium titanate (BT) nanoparticles (NPs) and P(VDF-TrFE) are used as the weight control terminal medium. The proposed device can withstand 10,000 bending cycles with 1.25% applied strain (Fig. 7B) and negligible change in synaptic weight and paired-pulse ratio under applying various strains (Fig. 7C). Furthermore, the work also demonstrated the reception and preprocessing of tactile information mimicking the biological Merkel cell and Merkel cell–neurite complex structure (Fig. 7D). A 2 × 2 TF-SAS array was constructed for the recognition of the number and intensity of mechanical external stimuli being applied. Figure 7E shows the 4 states of dipole generation and charge carrier transfer between the ferroelectric layer and the channel layer in the presence of tactile stimulation. At a fixed weight control terminal voltage (−3 V), the tactile stimuli (~0.3 kPa) alter the hole concentration in the channel due to dipole switching of the ferroelectric layer, and with variation in the amount of time pressure was kept on the weight control terminal, the PSC also changes (Fig. 7F). It was observed that the device can show considerable carrier retentivity even after discarding the tactile stimuli. The relative change in the PSC level with different pressures (left panel of Fig. 7G) and touch duration (right panel of Fig. 7G) showed that higher pressure and duration of touch can result in higher PSC and retentivity. The modulation of synaptic weight can be further controlled by controlling the composition of BT NPs, modulating the ferroelectric dipole switching intensity. With higher BT NPs being considered, the change in synaptic weight increases for both change in pressure (left panel of Fig. 7H) and touch duration (right panel of Fig. 7H). This finding underscores the potential of the device as an intelligent sensor memory for tactile recognition, opening new possibilities for its application in areas such as soft robotics and biocompatible interfaces.

### Chemoreception-functionalized SASs

**Fig. 8.**
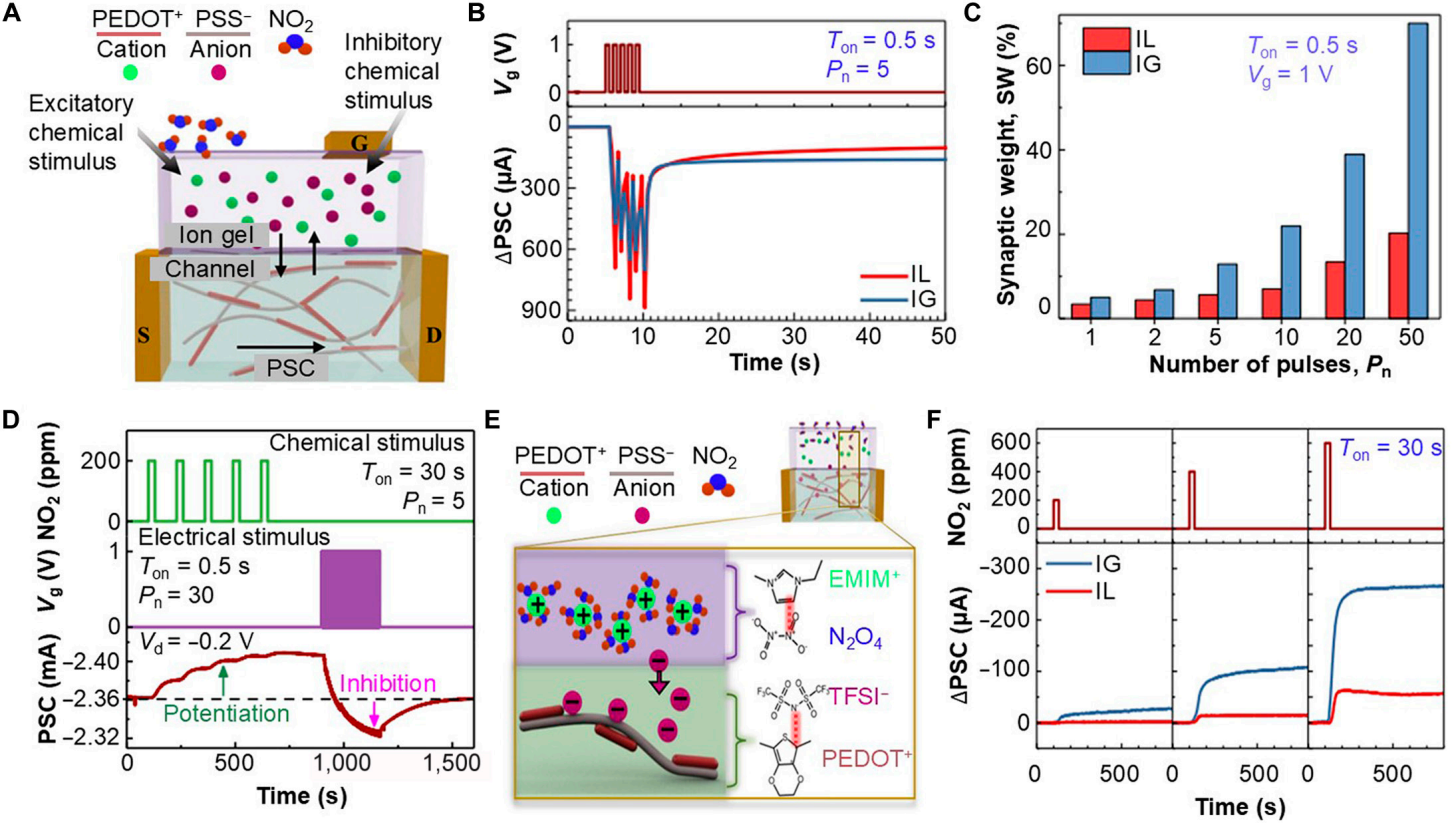
Functionalized soft chemical synapse (FSCS). (A) Schematic illustration of an FSCS with an ion-gel dielectric layer. (B) Variation of ΔPSC with ionic liquid (IL) and ion gel (IG), with the same gate electrical pulses, where *T*_on_ and *P*_n_ refer to pulse duration and pulse number, respectively. (C) Variation in synaptic weight with increasing number of pulses used with IL and IG, with 1-V gate pulses with a 0.5 s (*T*_on_) value. The postsynaptic terminal voltage is maintained at −0.2 V for all cases for spike-number-dependent plasticity (SNDP) properties. (D) Change in postsynaptic terminal current potentiation with chemical (NO_2_) and electrical (*V*_g_) stimuli applied as pulses for potentiation and depression, respectively. For PSC, 30 electrical pulses (*P*_on_) are used with a pulse of 0.5 s on-state (*T*_on_). (E) Underlying ion transfer mechanism in a chemical synaptic transistor in the presence of chemical stimuli. NO_2_ dimerizes to N_2_O_4_, which results in π–π interaction between [EMIM]^+^ and N_2_O_4_ and transfer of [TFSI]^−^ anions into the channel to interact with PEDOT^+^. (F) Variation of ΔPSC for both IL- and IG-gated devices, when the concentration of NO_2_ stimuli is kept at 200, 400, and 600 ppm [73].

Chemoreception-functionalized soft artificial synapses (CF-SASs) are gaining attention due to their ability to directly interact with biological systems, offering transformative potential for prosthetics and brain–machine interfaces. These interfaces employ advanced computational paradigms where hardware ANNs adapt autonomously through biofeedback. CF-SASs are unique in that they use chemical components, ions, molecules, and particles as input stimuli. Chouhdry et al. [73] emulated the potentiation and inhibition principle of the biological chemical synapse in the glomerulus (Mitral cell) within the olfactory bulb using an electrochemical transistor structure with chemosensory capability. The device uses a PEDOT:PSS channel with an electrolyte-gate dielectric layer, as shown in Fig. 8A. Figure 8B shows the spike-number-dependent plasticity of the proposed device to observe the change in the PSC in the absence of external chemicals. With the increased number of potentiation pulses, the percent change in synaptic weight increased, resulting in long-term memorization capability (Fig. 8C). Furthermore, the ionic liquid group in the electrolyte-gate dielectric layer ([EMIM]^+^ [TFSI]^−^) acts as a chemoreceptive layer with high carrier-inducing and gas solvent abilities. Figure 8D shows the interaction of molecular gas and electrolyte-gate dielectric, which generates weight control potential for potentiation application. Here, external chemical or gas molecules (NO_2_) can interact with the cations ([EMIM]^+^) and make them solvated. Additionally, the proposed chemoreceptive electrolyte-gate dielectric can be de-doped and doped through injection and extraction of the ions from the channel layer. In this case, the presence of NO_2_ and electrical stimuli (*V*_g_) results in potentiation and inhibition of the PSC, respectively. Figure 8E shows the dimerization of NO_2_ to form N_2_O_4_, which initiates the shift of [TFSI]^−^ anions into the channel to interact with PEDOT^+^. This chemical doping-induced change in the PSC is shown in Fig. 8F with 3 different concentrations of NO_2_. It was reported that a higher concentration of chemical exposure results in higher potential at the weight control terminal, resulting in higher doping levels and LTP. The long-term memorization implemented by the chemical stimulus can be erased by supplying weight control pulses, making the proposed device programmable for wearable and implantable chemosensory applications.

## Future Prospects and Challenges of Functionalized SAS Applications

Functionalized SASs offer several key benefits that traditional sensors and devices cannot effectively provide. The reduction of circuity in system design, minimized latency in signal transmission between computing and sensing components, ultralow energy (~15.38 aJ) per synaptic event, flexible device architecture, integration of neuromorphic computing within the sensing components, and potential for low-cost device manufacturing expand the scope of applications for functionalized SASs [27]. Building on these advantages, further research into functionalized SASs could explore a range of promising directions. First, PF-SASs involve expanding the frequency range of light and scaling up the synaptic arrays for autonomous vehicles and robotic vision systems. Additionally, artificial visual-perception systems based on PF-SASs offer promising options for integration into wearable and bioimplantable electronics. Second, for TF-SASs, the use of tribo-piezoelectric soft materials, such as PVDF, ZnO, BaTiO_3_, thin-film PZT:PDMS, and polyurethane, offers the ability for multimodal sensing that can show benefits in responsive and adaptive soft robotic systems developments, such as intelligent electronic skin in neurorobotics applications. Finally, the implementation of CF-SASs requires soft materials with chemoreceptor compliance for electrochemical sensing and potentiometric applications. Further investigation is necessary on soft materials such as PDMS, aqueous electrolytic dielectric solutions, and PEDOT:PSS ion gels, extending beyond olfactory and gustatory systems to electrochemical applications related to the human body and environmental sensing.

For clinical applications, such as neuroprosthetics and biomedical device design, functionalized SASs need to demonstrate biocompatible, miniaturized, scalable, and reliable competency. Materials like PEDOT:PSS and chitosan have shown promise due to their biocompatibility [96]. However, most organic semiconductors may degrade in vivo, leading to loss of functionality or adverse biological reactions [97]. An advanced encapsulation technique can be an option to protect the device from bodily fluids, but this still needs to be further investigated for potential application challenges [98–100]. Furthermore, the continuous interpretation of neural signals for practical interfacing with the human nervous system requires precise control, device integrity [101], and clear metrics to undergo rigorous testing and approval processes to ensure the host’s safety, data privacy, and long-term effects [102]. Table 4 shows important performance parameters for considering functionalized SAS devices for clinical applications. The challenges related to this unique clinical adoption involve searching for biocompatible polymers, composites, and bioinspired materials like conductive hydrogels [103]. Integrating fabrication techniques like 3-dimensional printing, nanolithography, and molecular self-assembly during the device fabrication process can help properly control the device size and scalability needs. Collaboration among multidisciplinary professionals is a prime need for in vivo testing and the iterative device improvement process, as well as for properly setting the metrics for embedding SASs and functionalized SASs into the human body.

**Table 4.** Performance parameters of functionalized SASs

Functionalized SAS type	Materials (semiconductor; dielectric)	Energy consumption	Response time	Biocompatibility	Biodegradability	In vivo application	Prosthetics application	Ref.
PF-SAS	SC-CNT; Al_2_O_3_/HfO_2_/AlOx NPs	~aJ to fJ (low)	μs to ms	Partial (Al_2_O_3_ biocompatible; CNTs conditional)	No	Potential (toxicity of CNTs must be addressed)	Potential	[107]
PF-SAS	MoS_2_; Al_2_O_3_/ZrO_2_/Al_2_O_3_;	-	μs to ms	Potential (materials generally biocompatible)	No	Potential	Potential	[108]
PF-SAS	MoSSe; Al_2_O_3_/BP QDs/Al_2_O_3_	-	μs to ms	Partial (BP QDs biodegradable and biocompatible)	Partial (BP QDs biodegradable)	Potential	Potential	[82]
PF-SAS	IGZO; chitosan/graphene oxide	-	μs to ms	Partial (chitosan biocompatible; GO under study)	Partial (chitosan biodegradable)	Potential	Possible	[111]
PF-SAS	P(IID-BT); P(VDF-TrFE)/P(VP-EDMAEMAES)	-	μs to ms	Potential (materials require further study)	No	Potential	Potential	[109]
PF-SAS	SC-SWCNT, CdSe/ZnS QDs PS-PMMA-PS/[EMIM][TFSI]	15.38 aJ/event	μs to ms	Limited (CdSe QDs are toxic)	No	Unlikely (due to toxicity)	Possible (external devices)	[27]
TF-SAS	Pentacene; P(VDF-TrFE)	-	μs to ms	Limited (pentacene not biocompatible)	No	Unlikely	Possible (external devices)	[64]
TF-SAS	Pentacene; BaTiO_3_ NPs/P(VDF-TrFE)	-	μs to ms	Limited (BaTiO_3_ NPs may be cytotoxic)	No	Unlikely	Possible (external devices)	[84]
CF-SAS	PEDOT:PSS PEGDA/[EMIM][TFSI]	-	μs to ms	Potential (PEDOT:PSS biocompatible)	Partial (PEGDA biodegradable)	Potential	Possible	[73]
CF-SAS	PEDOT:PSS; NaCl ion gel	-	μs to ms	Yes (PEDOT:PSS biocompatible)	Partial (depends on the ion-gel matrix)	Yes	Yes	[29]

To date, the functionalized SAS device technology is not mature for clinical practices. Some practical limitations, challenges, and trade-offs are crucial to understand the future implementation and integration into clinical aspects of these technologies. The soft or flexible nature of synaptic devices mostly comes from the organic materials being used; however, these materials could degrade over time and under harsh environmental conditions [104]. In addition, the in-memory sensing option offered by functionalized SASs needs careful attention, as the current literature lacks an in-depth investigation of the device’s performance in the presence of noise with or without strain. Moreover, future investigations in functionalized SASs require signal processing units and interfacing circuits and protocols, especially for wearable electronics and biosensing [105]. Finally, for wearable and implantable applications, proper material characterization is necessary to ensure the biocompatibility and nontoxicity of the material over the short and the long term [106]. Current techniques, such as encapsulation methods, require further refinement and specialized focus to effectively support SAS device operation.

## Conclusion

In the past decade, the realm of SASs has emerged as a field of profound interest, driven by its potential to revolutionize robotics, biosensing, and neuroprosthetics. Two critical attributes required for these SAS applications include consistent, flexible synaptic operation controllable through external stimuli and low energy consumption during operation. The choice of device materials is pivotal for achieving flexibility, while energy efficiency demands a multimodal approach to input stimuli. The functionalization of materials and device architectures is becoming increasingly crucial in facilitating both aspects of SAS operation.

This investigation provides a comprehensive overview of materials and device architectures for realizing SASs and functionalized SASs. Initially, it highlights the distinctive attributes of SASs in soft electronics, emphasizing their capacity to flex, bend, stretch, and adapt to uneven surfaces without compromising operational integrity. SASs primarily consist of organics, polymers, and nanomaterials, imparting the necessary softness to emulate biological systems. A shift in material choice from rigid inorganic materials to inherently flexible organic and low-dimensional materials for SAS realization is discussed. The research further elaborates on the operation of 3 well-known synaptic weight control mechanisms, floating-gate dielectric, ferroelectric-gate dielectric, and electrolyte-gate dielectric, along with their applications in neuromorphic image perception, tactile recognition, and dynamic programming. Subsequently, the operational mechanism of functionalized SASs, their device structure, and weight control mechanisms are explored. In functionalized SASs, weight control stimuli are directly integrated into the device, eliminating the need for external sensing devices like TENG or sensors. This results in enhanced multimodal sensing capabilities and improved energy efficiency. The functionalized operation with photonic, mechanical, and chemical stimuli is highlighted, offering prospects for future research and applications. Nevertheless, the research scope for other stimulus-based functionalized SASs remains limited and requires further investigation and development.

Applications of SAS necessitate nontoxic, flexible, energy-efficient, biodegradable, and biocompatible materials. The achievement of biofriendly flexible SASs holds the potential to revolutionize the biomedical and neuroelectronic fields, offering the promise of in vivo synaptic logic circuit implementation. Additionally, the susceptibility of SAS materials to environmental factors like heat and moisture raises questions about device reliability. These challenges underscore the importance of ongoing research and development to enhance the robustness, efficiency, and environmental resilience of SASs. This trajectory not only promises technological advancements but also envisions a symbiotic relationship between electronics and biological systems, fostering user-friendly, environmentally friendly, and human-centered electronic solutions.
